# Oxytocin Rapidly Changes Astrocytic GFAP Plasticity by Differentially Modulating the Expressions of pERK 1/2 and Protein Kinase A

**DOI:** 10.3389/fnmol.2017.00262

**Published:** 2017-08-15

**Authors:** Ping Wang, Danian Qin, Yu-Feng Wang

**Affiliations:** ^1^School of Basic Medical Sciences, Harbin Medical University Harbin, China; ^2^Department of Physiology, Shantou University Shantou, China

**Keywords:** astrocyte, glial fibrillary acidic protein, phosphorylated extracellular signal-regulated protein kinase1/2, oxytocin, protein kinase A, supraoptic nucleus

## Abstract

The importance of astrocytes to normal brain functions and neurological diseases has been extensively recognized; however, cellular mechanisms underlying functional and structural plasticities of astrocytes remain poorly understood. Oxytocin (OT) is a neuropeptide that can rapidly change astrocytic plasticity in association with lactation, as indicated in the expression of glial fibrillary acidic protein (GFAP) in the supraoptic nucleus (SON). Here, we used OT-evoked changes in GFAP expression in astrocytes of male rat SON as a model to explore the cellular mechanisms underlying GFAP plasticity. The results showed that OT significantly reduced the expression of GFAP filaments and proteins in SON astrocytes in brain slices. In lysates of the SON, OT receptors (OTRs) were co-immunoprecipitated with GFAP; vasopressin (VP), a neuropeptide structurally similar to OT, did not significantly change GFAP protein level; OT-evoked depolarization of astrocyte membrane potential was sensitive to a selective OTR antagonist (OTRA) but not to tetanus toxin, a blocker of synaptic transmission. The effects of OT on GFAP expression and on astrocyte uptake of Bauer-Peptide, an astrocyte-specific dye, were mimicked by isoproterenol (IPT; β-adrenoceptor agonist), U0126 or PD98059, inhibitors of extracellular signal-regulated protein kinase (ERK) 1/2 kinase and blocked by the OTRA or KT5720, a protein kinase A (PKA) inhibitor. The effect of OT on GFAP expressions and its association with these kinases were simulated by mSIRK, an activator of Gβγ subunits. Finally, suckling increased astrocytic expression of the catalytic subunit of PKA (cPKA) at astrocytic processes while increasing the molecular associations of GFAP with cPKA and phosphorylated ERK (pERK) 1/2. Upon the occurrence of the milk-ejection reflex, spatial co-localization of the cPKA with GFAP filaments further increased, which was accompanied with increased molecular association of GFAP with pERK 1/2 but not with cPKA. Thus, OT-elicited GFAP plasticity is achieved by sequential activation of ERK 1/2 and PKA via OTR signaling pathway in an antagonistic but coordinated manner.

## Introduction

Astrocytes are extensively involved in normal brain functions (Vasile et al., 2017) and neurological diseases (Pekny et al., 2016; Verkhratsky et al., 2016). However, cellular mechanisms underlying functional and structural plasticities of astrocytes are largely unknown, which requires further investigation. In astrocytes, functional roles of glial fibrillary acidic protein (GFAP), a major cytoskeletal element, have been identified in acute astrocyte plasticity (Langle et al., 2003; Sun et al., 2016). GFAP largely determines astrocytic morphology, which in turn changes astrocytic absorption of glutamate and ions, synaptic innervation and interactions between neighboring neurons (Hatton, 2004; Theodosis et al., 2008).

GFAP molecules are posttranslationally modified by a series of protein kinase. For example, protein kinase A (PKA) phosphorylated and destabilized GFAP *in vitro* (Inagaki et al., 1990; Heimfarth et al., 2016), and caused retraction of astrocytic processes (Hatton et al., 1991) from the surrounding of neurons. Increases in phosphorylated extracellular signal-regulated protein kinase (pERK) 1/2 elevated GFAP levels (Li D. et al., 2017), an effect similar to that of chronic cAMP/PKA activation (Hsiao et al., 2007). These findings thus highlight the dependence of GFAP metabolism on interactions between different protein kinases in astrocytes. However, these studies are mainly based on cell cultures or cell lines in a chronic time course, and it remains unclear how these kinases *in vivo* interact with each other in acute modulation of GFAP plasticity that is essential for both physiological regulation and pathogenesis of diseases (Wang and Hamilton, 2009; Wang and Parpura, 2016). Thus, determining the interaction between GFAP and these protein kinases in acute physiological processes is important to understand the cellular mechanisms that regulate GFAP/astrocytic plasticity.

In the study on glial neuronal interaction in the supraoptic nucleus (SON), GFAP plastic change occurred in concert with the change in oxytocin (OT) neuronal activity in the SON of lactating rats (Wang and Hatton, 2009). In the SON, OT can increase pERK1/2 expression; the distribution of pERK 1/2 was spatiotemporally associated with astrocyte/GFAP morphology (Wang and Hatton, 2007b), which is opposite to the effect of increased intracellular cyclic AMP (Hatton et al., 1991). Activation of OT receptors (OTRs) can trigger both pERK 1/2 and PKA signaling, and the functions of two kinases are mutually antagonistic in uterus (Zhong et al., 2003), which could also occur in the SON. Thus, we hypothesized that both pERK 1/2 and PKA are involved in GFAP plasticity while they function differently in OT-elicited astrocytic plasticity in the SON.

To test this hypothesis, we used OT-evoked GFAP plasticity in male rats first to study the contribution of these two signaling pathways to GFAP plasticity. We found that astrocytes in the SON expressed OTRs and OT could depolarize astrocyte membrane potential while reducing GFAP expression via OTRs. Effects of OT on GFAP plasticity and on the absorption of an astrocyte-specific peptide were differentially modulated by PKA and pERK 1/2. The effect of OT on GFAP expressions and its association with pERK 1/2 and PKA were simulated by an activator of Gβγ subunits. In lactating rats, suckling of pups increased astrocytic expression of PKA at astrocytic processes, which was accompanied with increased molecular associations between GFAP with PKA and pERK 1/2. When the milk-ejection reflex occurred, catalytic subunit of PKA (cPKA) co-localized with GFAP filaments further increased, and the molecular association of GFAP with pERK 1/2 but not with cPKA was also increased. Along with findings of pERK 1/2 involvement in GFAP plasticity in the SON (Wang and Hatton, 2009), the present results highlight that OT-elicited GFAP plasticity is associated with sequential activation of ERK 1/2 and PKA downstream to OTR signaling in an antagonistic but coordinated manner.

## Materials and Methods

Experiments were performed using adult male (42–60 days old) and lactating female Sprague-Dawley rats. This study was carried out in accordance with the recommendations of NIH guidelines. The protocol was approved by Institutional Animal Care and Use Committees of the University of California-Riverside and Harbin Medical University, respectively.

### Drugs, Reagents and Antibodies

OT, β-Mercapto-β, [β-cyclopentamethylene-propionyl^1^, O-Me-Tyr^2^, Orn^8^]-OT (OTR antagonist, OTRA), vasopressin (VP), isoproterenol (IPT, activator of β-adrenoceptor and PKA), KT5720 (inhibitor of PKA), PD98059 and U0126 (inhibitors of ERK 1/2 kinase), U73122 (inhibitor of the coupling of G protein-phospholipase C activation), tetanus toxin and others were from Sigma except as otherwise noted. Myristoylated G-protein βγ-binding peptide [myristoyl-SIRKALNILGYPDYD (mSIRK)] was from EMD Biosciences. Reagents for Western blots were from GE Healthcare. Primary antibodies were from Santa Cruz Biotechnology except for mouse anti-pERK 1/2 (Cellular Signaling), mouse anti-OT neurophysin (NP) and VP-NP (Dr. H. Gainer, NIH, Bethesda, MD, USA). Bauer peptide (β-Ala-Lys-N_ε_-AMCA) was provided by Dr. K. Bauer (Max-Planck-Institut für experimentelle Endokrinologie, Hannover, Germany). Secondary antibodies were from Thermo Fisher Scientific.

### Slice Preparation

Rats were decapitated; the brain was quickly removed and immersed in ice-cold slicing solution that was oxygenated through bubbling with a compressed gas mixture of 95% O_2_/5% CO_2_. The slicing solution contained 1/3 of 10% sucrose and 2/3 regular artificial CSF. Then, coronal slices (200 μm thick) were obtained from the SON as previously described (Wang and Hatton, 2009). Slices were pre-incubated at room temperature (21–23°C) for 1 h in oxygenated regular artificial CSF before drug treatment or application of other procedures. The regular artificial CSF contained (in mM): 126 NaCl, 3 KCl, 1.3 MgSO_4_, 2.4 CaCl_2_, 1.3 NaH_2_PO_4_, 26 NaHCO_3_, 10 Glucose, 0.2 ascorbic acid, pH 7.4 and 305 mOsm/kg, oxygenated with 95% O_2_/5% CO_2_. The slice was then randomly assigned to groups for immunostaining or protein analyses.

In suckling experiments, lactating rats with 4 h separation from pups were allowed to suckling of 10 pups for 0–30 min as previously described (Wang and Hatton, 2009). According to the occurrence of the milk-ejection reflex, brains were collected in three groups, i.e., non-suckling, suckling (for 5–10 min before the first milk ejection), and milk-ejection reflex (suckling until the occurrence of the third or fourth milk ejections). Brain were fixed immediately without pre-incubation or homogenized to obtain protein lysates.

### Immunocytochemistry and Confocal Microscopy

Immunostaining was performed based on our earlier reports (Wang and Hatton, 2009) with minor modifications. In brief, slices were permeated with 0.3% Triton X-100 in 0.1 M PBS for 30 min, and non-specific binding was blocked with 0.3% gelatin-PBS. The slices were then incubated overnight at 4°C with primary antibodies against goat or mouse OT-NP (1:400) and VP-NP (1:400), goat or mouse GFAP (1:300), mouse pERK 1/2 (1:1000), rabbit cPKA (1:250) and goat OTRs (1:250). After rinsing, the slice was incubated with species-matched fluorescent donkey anti-goat/mouse/rabbit antibodies (Alexa Fluor^®^ 647/555/488, 1:1000) for 1.5 h at room temperature (22–24°C). Finally, Hoechst stain (0.5 μg/ml for 15 min) was used to label nuclei.

For each treatment, 6–12 pieces of slices from the middle part of the SON of 3–6 rats were imaged at high magnification (630×), 10–20 μm from the surface using a laser scanning confocal microscope (Leica TCP SP2 or Zeiss LSM510). Multiple fluorophores were imaged sequentially, and distribution pattern and colocalization of different molecules were analyzed. To avoid false positive or negative results of immunostaining, serial dilutions of the primary antibody, staining with pre-absorbed (immune-neutralization) primary antibody, no-primary and no secondary antibody controls were applied.

### Western Blots and Co-Immunoprecipitation (Co-IP)

Methods for protein analysis were the same as previously reported (Wang et al., 2013a,b). In brief, hypothalamic slices from three to six rats were obtained as described above. SONs were punched out and then lysed. The lysates were centrifuged to remove insoluble components before protein levels were quantitated using a plate reader. Protein aliquots (60 μg) were loaded and separated on 10% SDS-PAGE gels, and then transferred onto polyvinylidene difluoride membranes. After blocking with 5% milk solids (or 1% gelatin for detecting primary antibodies from goat) for 1 h at room temperature, membranes were incubated with mouse or goat anti-GFAP, rabbit or goat anti-OTRs, rabbit anti-cPKA and mouse anti-pERK 1/2 (all 1:500) overnight at 4°C. To calibrate protein levels, mouse anti-tubulin (1:300), rabbit anti-actin (1:500) or anti-total ERK2 (tERK2, 1:1000) were also detected (1 h at room temperature). Bands were visualized using horseradish peroxidase-conjugated secondary antibodies and an enhanced chemiluminescence system (Tanon 5200, Shanghai). Data are reported for 3–6 replicates.

For Co-IP experiments, SON lysates were precleared with protein G agarose beads to reduce nonspecific binding. Mouse anti-GFAP (1.5 μg/7.5 μl) was then added to the lysates (1500 μg/500 μl protein) to form an immunocomplex and incubated overnight at 4°C. Immunocomplexes were captured by adding 50 μl of protein G agarose bead slurry and gently rocking for 2 h at 4°C. They were then collected, washed and resuspended in 50 μl 2× Western blotting sample buffer, and boiled for 10 min to dissociate proteins from the beads. Target proteins were then detected using Western blotting.

### Patch-Clamp Recordings

Patch-clamp recording procedures for SON were similar to those described previously (Wang and Hatton, 2004). Briefly, after pre-incubation in the regular artificial CSF, slices were incubated in artificial CSF containing Bauer peptide that is fluorescent and can be selectively taken up by astrocytes via peptide transporter PepT2 (Dieck et al., 1999) at 35°C for 2–4 h, with or without tetanus toxin (1 nM) to block synaptic vesicle release. Whole cell patch-clamp recordings were obtained from fluorescent cells visualized using an epifluorescence microscope using an Axopatch 200B amplifier or Multiclamp 700B amplifier (Molecular Devices). The pipette solution for recording SON astrocytes contained (in mM): 145 K-gluconate, 10 KCl, 1MgCl_2_, 10 HEPES, 1 EGTA, 0.01 CaCl_2_, 2 Mg-ATP, 0.5 Na_2_-GTP, pH 7.3, adjusted with KOH. Signals that were filtered, sampled at 5 kHz, and analyzed offline using Clampfit 10 software (Molecular Devices). Exemplary cells were also tested their voltage-current relationship and examined in immunohistochemistry after drug tests to verify their astrocytic nature.

### Real Time Imaging of Astrocytes

To link modulating effects of OT on GFAP plasticity to astrocytic functions, slices were incubated with the artificial CSF containing 0.1 μM Bauer peptide as described above. Then, astrocytic somata in the ventral glial lamina (VGL) of the SON were focused and their fluorescence intensity was captured through a Microfire Camera in single frame following a brief (20–30 s) exposure to UV light. Drugs were bath-applied for 15 min, and images were captured immediately before, 5 min and 15 min after drug application, respectively. Preparations were fixed *in situ* at the end of observations for further analyzing images by using confocal microscopy as previously reported (Wang and Hatton, 2009).

### Data Analysis

Methods for analyzing data of immunocytochemistry, Western blots and patch-clamp recordings were modified from our previous experiment (Wang and Hatton, 2009). To evaluate GFAP levels, the fluorescence intensity in each channel was normalized to a standard curve (1–256) to allow for comparison between different experiments. The background fluorescence level was set as 1 through minimum baseline correction using Leica LCS Lite or ZeissLSM software and maximum intensity was set at 256. To assay GFAP expression in single scan-based confocal image, whole frame of the image was compared on the same background level of fluorescence intensity. The efficiency of this single sectioned image in reflecting GFAP plasticity had been validated by Z-stack scanning (0.5 μm/section) and fluorescent microscopy as described previously (Wang and Hatton, 2009). The increase or decrease in the expression level of GFAP was defined as a change more than 20% from the control. In analyzing the expression of GFAP filaments, they were first distinguished from the cell body by its extending from but not surrounding “astrocytic nucleus” and appearing thread-like rather than circular morphology. The diameter of GFAP filaments was measured at five sub-sections including those near each of the four corners and the center in a square frame and their average was used to represent the diameter of a section, which was applicable in 95% of the images.

In determining different components of GFAP protein, the monomers were identified by the appearance of a single 50 KDa band and the fragments included the diffuse bands below 50 KDa and above 35 KDa. In patch-clamp recordings, the membrane potential was an average level of 1 min before and 1 min (9th–10th min) after drug application. In analyzing the fluorescent intensity of Bauer peptide-loaded astrocytes, background fluorescence was subtracted from the dorsal portion of the SON that did not show clear astrocyte soma; changes in fluorescence were calculated by comparing the intensity after drugs with those before drugs.

Student’s *t*-test and ANOVA were used for statistical analyses where appropriate, as instructed by SigmaStat 12 program, and *p* < 0.05 was considered significant. When abnormal distribution or large variances appeared, square-root transformations (for some Co-IP studies with *n* = 3) were applied to minimize the influence of individual data points on the evaluation of whole significance level. All measures were expressed as mean ± SEM in percentage of control values, or as otherwise noted.

## Results

In this study, we first examined the features of OT-evoked GFAP plasticity of astrocytes in the SON of male rats and its dependence on OTR-Gβγ signaling. Next, we explored the roles of pERK 1/2 and PKA in the GFAP plasticity and their association with astrocytic absorption of fluorescent peptide. Lastly, we linked the roles of pERK 1/2 and PKA to suckling-evoked GFAP plasticity of the SON in lactating rats to verify the applicability of identified features of kinase modulation of acute GFAP plasticity.

### OT Changed GFAP Expression in the SON of Male Rats

**Figure 1:**
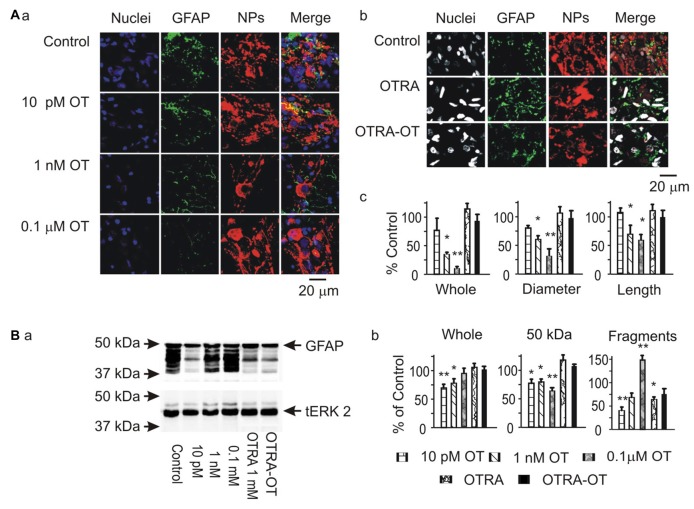
Effects of oxytocin (OT) on glial fibrillary acidic protein (GFAP) plasticity in the supraoptic nucleus (SON) in brain slices from male rats. **(A)** Effects of OT (10 pM, 1 nM and 0.1 μM for 30 min) and OT receptor antagonist (OTRA, 1 μM for 30 min) on GFAP expression in confocal images. **(Aa,Ab)** Staining of nuclei, GFAP, neurophysins (NPs) and their merges (left to right). **(Ac)** Summary graphs showing the intensity of GFAP filaments relative to the control in whole panel, diameters and lengths of the filaments. Note that: *P < 0.05 and **P < 0.01 compared to the control (*n* = 6) in ANOVA with Holm-Sidak comparisons. **(B)** GFAP expression in Western blots. **(Ba)** Bands (left to right) show GFAP proteins (top) for control, 10 pM OT, 1 nM OT, 0.1 μM OT, 1 μM OTRA and 0.1 nM OT after OTRA for 30 min, respectively. Bands at the bottom are total extracellular signal regulated protein kinase 2 (tERK 2), serving as loading controls for corresponding treatments (*n* = 6, compared in ANOVA). **(Bb)** Summary graphs showing GFAP relative to control in whole bands, bands at 50 kDa (full size), and their smaller fragments, respectively.

To analyze the potential modulatory effects of signaling molecules on GFAP plasticity in the SON, we first examined effects of OT on GFAP expression in brain slices from six male rats. The results showed that OT, at 10 pM, 1 nM and 0.1 μM for 30 min, concentration-dependently reduced GFAP levels in the SON in confocal microscopy. As shown in Figure 1A, before OT treatment (Control), GFAP staining was less compact in perinuclear areas of astrocytes while GFAP filaments were in clear and rich bundles. Accompanying with the general reduction of GFAP, the length and diameter of GFAP filaments were reduced significantly by OT, whereas GFAP staining was increased at the somata (Figure 1Aa). Pretreatment of the slices with [β-Mercapto-β, β-cyclopentamethylene-propionyl^1^, O-Me-Tyr^2^, Orn^8^]-OT blocked OT-elicited GFAP reduction (Figure 1Ab). In Western blots, different concentrations of OT differentially influenced the expressions of different components of GFAP protein (Figure 1B). OT at 10 pM for 30 min significantly decreased 50 kDa GFAP protein and smaller GFAP fragments. At 1 nM and 0.1 μM, OT still decreased 50 kDa GFAP but significantly increased small GFAP fragments. The concentration-dependent effects of OT on GFAP plasticity were exhibited as the increases in GFAP fragments rather than 50 KDa components. Moreover, pretreatment of slices with the OTRA blocked this OT effect (Figure 1Ba). These results summarized in Figures 1Ac,Bb are consistent with the effect of OT on GFAP plasticity in lactating rats (Wang and Hatton, 2009).

In the SON, there are two major forms of astrocytes, radial glia-like morphology in the VGL, and stellate morphology in the somatic area (Israel et al., 2003). Here, we analyzed OT effects on GFAP expression in these two forms of astrocytes by observing GFAP expression with nuclear staining. The result showed that OT-reduced GFAP staining was significantly stronger (*n* = 6, *P* < 0.05 by paired *t*-test) in the dorsal SON (34.8 ± 7.4% of control) than in the ventral SON (72.7 ± 8.6% of the control by paired *t*-test). This is in agreement with the finding that osmotic stimulation causes glial retraction around dorsally located OT neurons but not ventrally located VP neurons in the SON (Chapman et al., 1986).

### Specificity of OT Actions on GFAP Expression in Astrocytes

**Figure 2:**
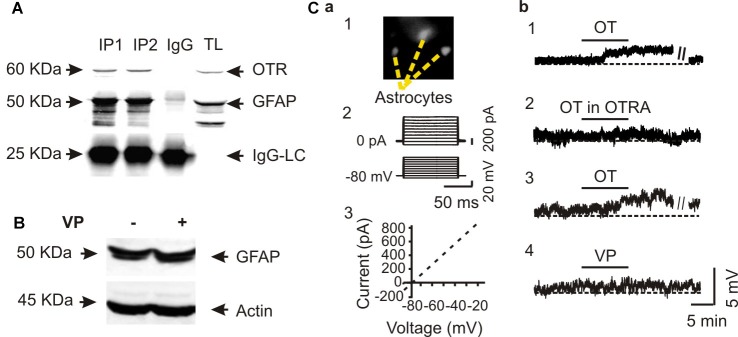
Specific effects of OT on GFAP plasticity. **(A)** Co-immunoprecipitation (Co-IP) of GFAP with OTRs: Western blotting of OTRs (top panels), immunoprecipitated GFAP (the middle panels and IgG light chains, IgG-LC, bottom) in two duplicates (IP1 and IP2) along with the loading of total lysis (TL). **(B)** Western blots showing that effect of vasopressin (VP, 0.1 nM, 30 min) on the expression of GFAP in SON protein lysates. **(C)** Patch-clamp recordings showing effects of OT on the membrane potential of astrocytes in brain slices. **(Ca)** Identification of astrocytes by Bauer peptide loading **(Ca1)** and by the linear current-voltage relationship **(Ca2,Ca3)**. **(Cb1)** Depolarizing effects of OT (0.1 nM, 10 min) on astrocytes in the SON. **(Cb2)** Effects of OTRA (1 μM, 10 min before OT) on OT-evoked depolarization. **(Cb3,Cb4)** Effects of OT **(Cb3)** and VP **(Cb4)** at 0.1 nM for 10 min on the membrane potentials after pretreatment with tetanus toxin (10 nM, 2–4 h). Other annotations refer to Figure 1.

To verify the specificity of OT actions on astrocytes, we performed Co-IP of GFAP with OTRs in three rats. The result showed a clear molecular association between GFAP and OTRs (Figure 2A). This result is consistent with our previous finding in immunocytochemistry that OTRs were present in GFAP-positive astrocytes in the SON (Wang and Hatton, 2006).

Next, we tested effects of VP, a nonapeptide in the SON structurally similar to OT, on GFAP protein levels. In contrast to the strong effects of OT on GFAP level in the SON, VP (0.1 nM, 30 min) did not significantly influence the expression of GFAP (101.0 ± 17.3% of control, *n* = 5, *P* > 0.05 by pared *t*-test) in Western blots (Figure 2B). Moreover, in Bauer peptide (20 μM)-loaded astrocytes in brain slices (Figure 2Ca1) that possessed linear voltage-current relationship (Figures 2Ca2,Ca3) we found that OT (0.1 nM, 5–10 min) significantly depolarized astrocytic membrane potentials (67.4 ± 2.3 mV vs. 63.5 ± 1.8 mV at 10 min, *n* = 7, *P* < 0.01 by paired *t-test*; Figure 2Cb1), which was blocked by pretreatment of slices with OTRA (67.1 ± 3.0 mV vs. 67.0 ± 3.0 mV at 10 min, *n* = 7, *P* > 0.05 by paired *t*-test; Figure 2Cb2). By contrast, pretreatment of astrocytes in three slices with tetanus toxin (10 nM, 2–4 h) did not block the depolarizing effects of OT (66.0 ± 3.0 mV vs. 62.3 ± 3.3 mV at 10 min, *n* = 6, *P* < 0.01 by paired *t*-test; Figure 2Cb3) while VP had no significantly effect on the membrane potential (67.0 ± 2.4 mV vs. 67.1 ± 2.2 mV at 10 min, *n* = 6, *P* > 0.05 by paired *t-test*; Figure 2Cb4). These results are consistent with our previous finding in lactating rats (Wang and Hatton, 2009) that the presence of tetanus toxin did not influence OT-elicited GFAP reduction.

### Signaling Cascades Mediating OT-Evoked GFAP Plasticity

**Figure 3:**
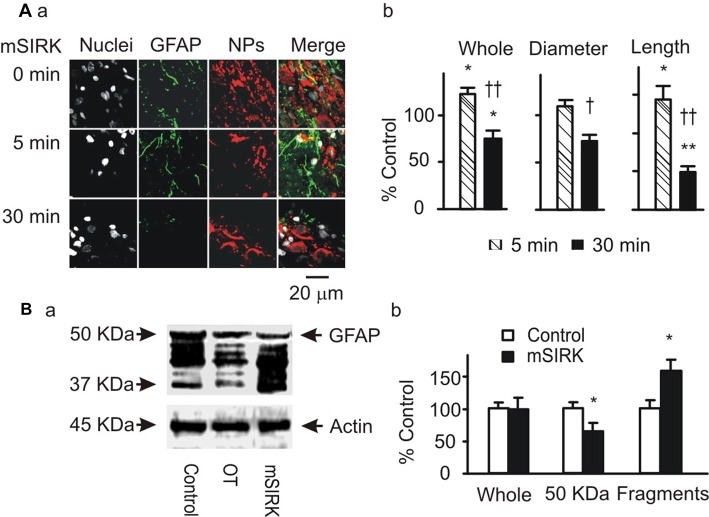
Roles of Gβγ subunits in OT-elicited GFAP plasticity. **(A)** Exemplary confocal images showing (left to right) nuclei, GFAP, OT plus VP NPs and their merges at 0 min, 5 min and 30 min after treatments with myristoyl-SIRKALNILGYPDYD(mSIRK; 0.5 μM), respectively **(Aa)**. **(Ab)** Summary graphs showing mSIRK effects on different components of GFAP. *P < 0.05 and **P < 0.01 compared to 0 min controls; ^†^*P* < 0.05 and ^††^*P* < 0.01 compared to mSIRK at 5 min. **(B)** Western blot showing effects of mSIRK on different bands of GFAP proteins (**Ba**, relative to the effect of OT) and the summary graphs of the mSIRK effect **(Bb)**. Other annotations refer to Figure 1.

Gβγ signaling cascade was the major approach mediating OT modulation of OT neuronal activity (Wang and Hatton, 2007a,b) and could also be the mediator of OT modulation of GFAP plasticity. To clarify this issue, we first examined GFAP expression after treatment of slices with mSIRK, an activator of Gβγ subunits. As shown in Figure 3A, mSIRK (0.5 μM) dually changed GFAP expression significantly (*n* = 6, *P* < 0.05 by ANOVA) in a time-dependent manner. At 5 min after mSIRK treatment, GFAP filaments were increased significantly (*n* = 6, *P* < 0.05), which then significantly reduced at 30 min (*n* = 6, *P* < 0.05). In Western blots, mSIRK (0.5 μM, 30 min) significantly reduced 50 kDa GFAP bands but increased GFAP fragments (Figure 3B), an effect similar to that of OT. These findings are in agreement with the time-dependent effect of OT on GFAP plasticity in the SON of lactating rats (Wang and Hatton, 2009).

**Figure 4:**
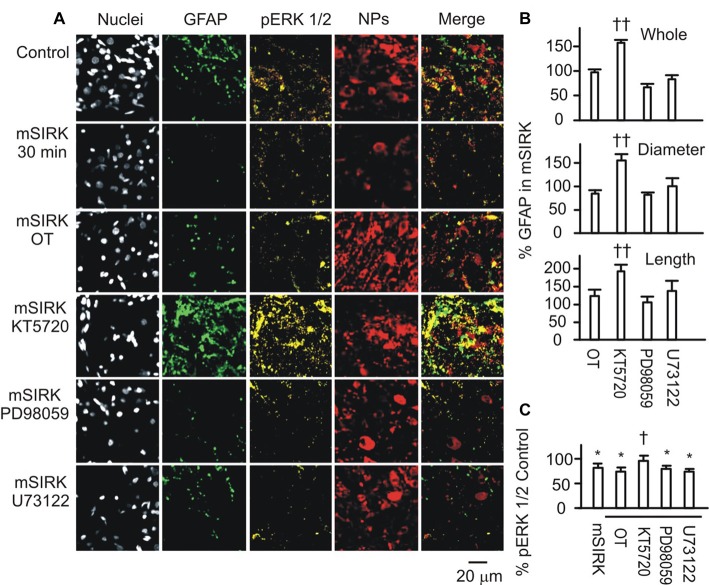
Roles of OTR-associated signals in Gβγ subunit-modulated expressions of GFAP and phosphorylated extracellular signal-regulated protein kinase (pERK) 1/2. **(A)** Confocal images (left to right) show staining for nuclei, GFAP, pERK 1/2, NPs and their merges. From top to the bottom, images were acquired in control, mSIRK (0.5 μM, 30 min) and mSIRK following 30 min pretreatment with OT, KT5720 (10 μM), PD98059 (20 μM), or U73122 (10 μM), respectively. **(B)** Summary graphs of GFAP expression in whole field view, the diameter, or length of GFAP filaments relative to mSIRK alone. **(C)** Summary graphs of pERK 1/2 under different conditions. Note that, *P < 0.05 compared to the control; ^†^*P* < 0.05 and ^††^*P* < 0.01 compared with mSIRK alone. Other annotations refer to Figures 1, 3.

Next, we observed effects of blocking downstream signaling of Gβγ subunits on mSIRK-evoked GFAP plasticity. As shown in Figure 4A, mSIRK (0.5 μM, 30 min)-evoked reduction of GFAP and pERK 1/2 immunostaining was differentially influenced by pretreatments of the slices (*n* = 6) with OT (1 nM, 30 min), KT5720 (10 μM), PD98059 (20 μM) and U73122 (10 μM). Compared to the effect of mSIRK only on GFAP filaments, addition of OT, PD98059 or U73122 did not significantly influence GFAP levels; however, the addition of KT5720 significantly increased both GFAP and pERK 1/2 expressions. This finding is in agreement with previous finding that Gβγ signaling cascade including pERK 1/2 was the major mediator of OT effect in the SON (Wang and Hatton, 2007b) and that PKA antagonized pERK 1/2 signaling (Zhong et al., 2003) and promoted GFAP depolymerization (Inagaki et al., 1990; Heimfarth et al., 2016).

### Roles of pERK 1/2 and PKA in OT-Evoked GFAP Plasticity

**Figure 5:**
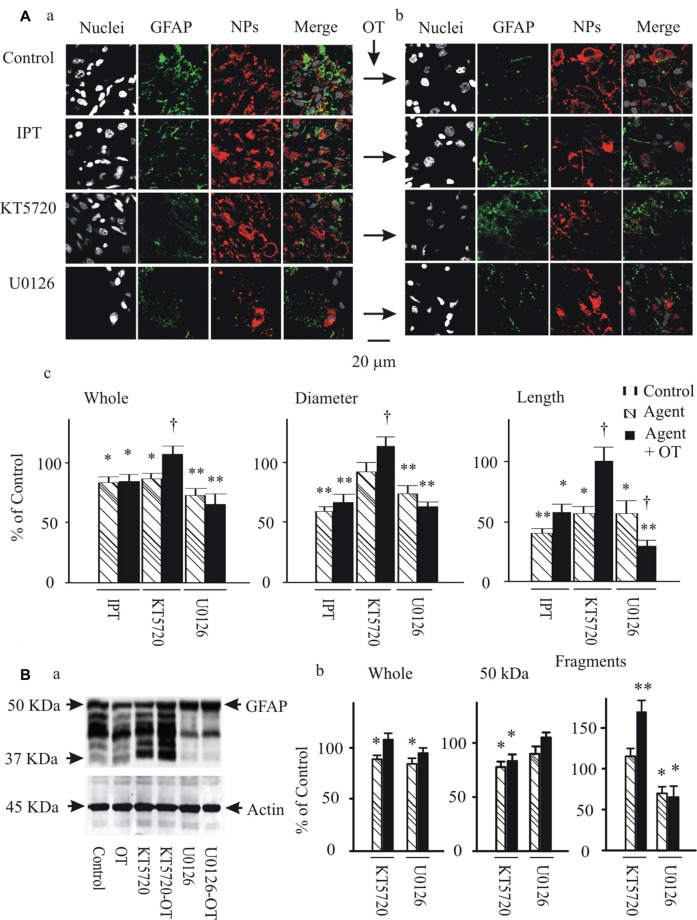
Effects of modulating protein phosphorylation on OT-evoked GFAP reduction. **(A)** Exemplary confocal images showing (left to right) nuclei, GFAP, NPs and their merges before **(Aa)** and after **(Ab)** application of OT (1 nM, 30 min). Note: IPT, isoproterenol (10 μM), KT5720 and U0126 (1 μM). OT was added 30 min after these agents. **(Ac)** Summary graphs showing relative intensity of GFAP staining under different conditions. *P < 0.05 and **P < 0.01 compared to the control; ^†^*P* < 0.05 compared with agents only. **(B)** GFAP expressions in Western blots. **(Ba)** Bands (left to right) showing GFAP proteins (top) in control, 1 nM OT, KT5720, KT5720 plus OT, U0126 and U0126 plus OT, respectively. Bands at bottom are actin, serving as loading controls for corresponding treatments (*n* = 6). **(Bb)** Summary graphs showing different components of GFAP relative to controls. Other annotations refer to Figures 1, 4.

To further test if and how pERK 1/2 and PKA were involved in OT-elicited GFAP plasticity, we observed effects of OT (1 nM) on GFAP plasticity after changing the activity of pERK 1/2 or PKA 30 min before OT application. Immunostaining and confocal microscopy (Figure 5Aa) showed that the staining of GFAP was substantially (*p* < 0.05 or 0.01, *n* ≥ 6) reduced by IPT (10 μM) and U0126 (1 μM) respectively, and slightly but significantly (*P* < 0.05) reduced by KT5720 (10 μM). The effects of IPT and U0126 on GFAP filaments were on both somata and processes, while the effect of KT5720 was on the processes only. Moreover, addition of OT (Figure 5Ab) did not significantly influence the actions of IPT or U0126, but reversed the effect of KT5720. The effects of these agents on OT-evoked GFAP plasticity are summarized in Figure 5Ac and are consistent with their effects on mSIRK-evoked GFAP plasticity.

Following the immunohistochemical experiments, Western blots were performed to detect GFAP protein after KT5720 and U0126 treatments with and without the presence of OT in brain slices from 6 rats. As shown in Figure 5Ba, KT5720 significantly decreased the 50 kDa GFAP bands; addition of OT strongly increased both 50 kDa and the fragments of GFAP proteins. By contrast, U0126 significantly reduced small GFAP bands but enhanced 50 kDa bands, which were not significantly changed by addition of OT (see summary graph in Figure 5Bb). These results are in agreement with the finding in confocal microscopy.

### Effects of GFAP-Modulating Agents on Astrocytic Uptake/Retention of Bauer Peptide

**Figure 6:**
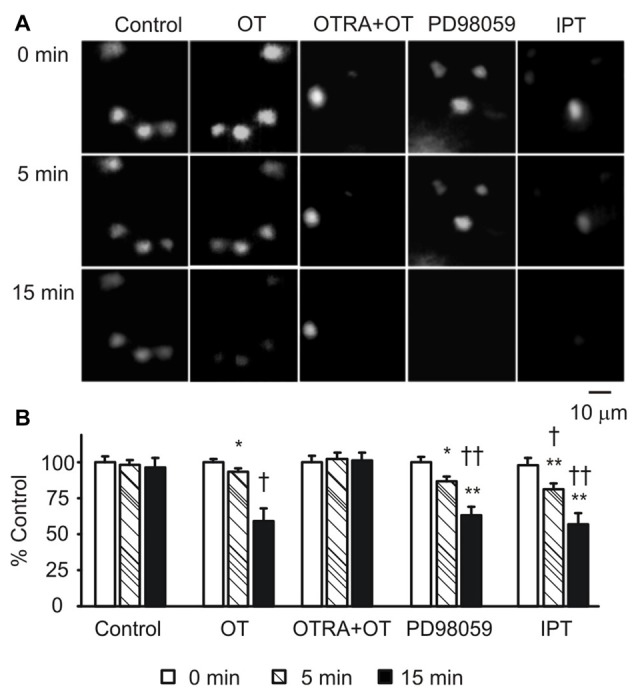
Effects of GFAP-modulating agents on astrocytic uptake of Bauer peptide. **(A)** Exemplary fluorescence images showing temporal changes of fluorescence intensity of astrocytes that were preloaded with Bauer peptide (20 μM, 2–4 h at 35°C) in the SON. From left to the right the columns were control, OT (1 nM), OTRA (1 μM, from 5 min before OT) plus OT, PD98059 (20 μM) and IPT (10 μM), respectively. **(B)** Summary graphs. Note that, *P < 0.05, **P < 0.01 compared to the controls; ^†^*P* < 0.05 and ^††^*P* < 0.01 compared with those at 5 min. Other annotations refer to Figures 1, 4.

To link the GFAP-modulating effects of pERK 1/2 and PKA with astrocytic functions, we observed effects of OT, PD98059 and IPT on the fluorescence intensity of astrocytes after loading Bauer peptide in brain slices (*n* = 6) from three rats. As shown in Figure 6A, OT significantly reduced the fluorescence intensity after 15 min treatment compared to the time-matched controls, effects of which were mimicked by using PD98059 and IPT, and blocked by pretreatment of the slices with OTRA from 5 min before these agents (see Figure 6B for summary).

### Effects of Suckling Stimulation on cPKA Expression and the Molecular Associations of GFAP with pERK 1/2 and PKA

**Figure 7:**
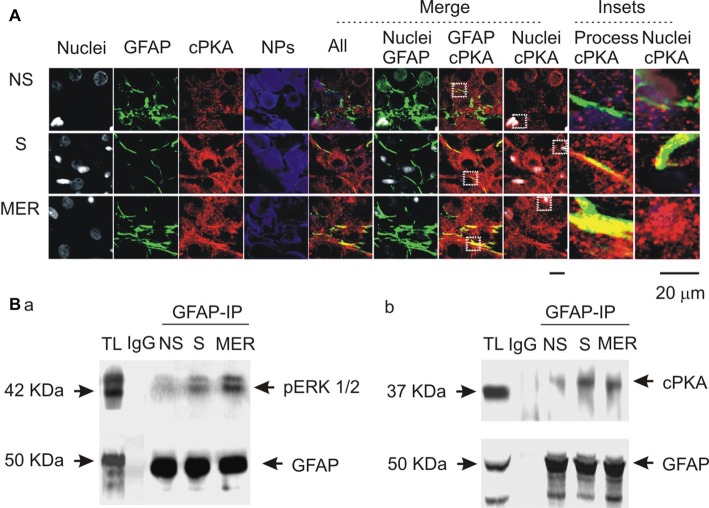
Spatial and molecular associations of GFAP with protein kinases. **(A)** Exemplary confocal images showing effects of suckling at different stages on the expression of catalytic subunit of protein kinase A (cPKA). Images from left to the right showing nuclei, GFAP, cPKA, NPs, the merges of all channels, GFAP with nuclei, GFAP with cPKA, nuclei with cPKA, and the insets expanded from white dashed squares in corresponding merged channels. **(B)** Molecular association of GFAP with pERK 1/2 **(Ba)** and with cPKA **(Bb)** at different stages of suckling as indicated. Note that, NS, non-suckling; S, suckling; MER, immediately after the milk-ejection reflex. Other annotations refer to Figures 1, 4.

To link the differential GFAP-modulating effects of the kinases to astrocytic functions, we further examined cPKA expressions at different stages of suckling in immunocytochemistry in three sets of lactating rats, based on previous observation of pERK 1/2 (Wang and Hatton, 2009). As shown in Figure 7A, suckling significantly increased cPKA expression in whole SON. In astrocyte profiles, cPKA was markedly increased at the processes as indicated by its heavy overlapping with GFAP. The occurrence of the milk-ejection reflex further increased the co-localization of cPKA with GFAP filaments, but reduced cPKA expression at neuronal profiles from the peak at the initial stage of suckling. Further examination of the molecular association of cPKA and pERK 1/2 with GFAP in Co-IP experiments (Figure 7B) revealed that molecular association of GFAP with pERK 1/2 (204.0 ± 23.6% of control, *n* = 3, *P* < 0.05) increased significantly (*n* = 3, *P* < 0.05) at the initial stage of suckling; the Co-IP of cPKA with GFAP was increased in all the three cases although the increase in the average level (178.2 ± 30.8% of control, *P* = 0.053) did not reach statistical significance due to the relatively low power. Upon the occurrence of the milk-ejection reflex, the association of GFAP with pERK 1/2 (431.4 ± 66.0% of control, *n* = 3, *P* < 0.05 compared to those of the control and during suckling) was further increased (Figure 7Ba), whereas the association between GFAP and cPKA was decreased significantly from that during suckling (104.5 ± 54.9% of the control, *n* = 3, *P* < 0.05 compared to that during suckling; Figure 7Bb). These results are in agreement with the findings in pharmacological manipulation in the males.

## Discussion

In the present study, we found that OT-evoked reduction of GFAP filaments depends on an antagonistic but coordinated pERK 1/2 and PKA interaction, downstream to OTR-Gβγ signaling. Upregulation of pERK 1/2 facilitates GFAP polymerization and PKA suppresses this polymerization while providing a basal condition for pERK 1/2 to function. Moreover, the two kinases work in coordination to localize GFAP at different compartments of astrocytes. This cellular signaling process is common between male and lactating female rats, likely contributing to astrocytic modulation of OT neuronal activity under diverse physiological conditions.

### Methodological Consideration

To study astrocytic plasticity, classically electron microscopy is the first choice. However, at this level, GFAP is often absent from fine astrocytic processes around synapses and neuronal somata (Theodosis et al., 2008). Thus, despite the power of electron microscopy in revealing detailed morphological features of astrocytic processes and their association with particular neuronal and astrocytic populations, it is not more helpful in studying GFAP plasticity than confocal microscopy plus proteomic analyses. *In situ* observation of GFAP plasticity using rats that express luciferase under GFAP promoters to image GFAP can provide a real-time image of GFAP filaments. However, *in vivo* imaging of fluorescent GFAP in the SON is limited by the anatomical complexity in exposing the SON surgically. In the present study, combining confocal microscopy with protein analysis, patch-clamp recordings, and fluorescence detection of astrocyte-specific peptide in brain slices could not only reveal OT-elicited GFAP reduction and the underlying molecular mechanisms, but also establish a functional association of GFAP with astrocytic plasticity as discussed below.

### OTR Mediation of OT-Evoked GFAP Reduction

GFAP monomers and filaments were reduced upon exposing to OT in males via activating OTRs on astrocytes in the SON. This finding is in agreement with previous reports that suckling acutely reduced GFAP expression via activation of OTRs (Wang and Hatton, 2009) and that a global reduction of GFAP expression occurred at the late pregnancy and during lactation in the SON (Perlmutter et al., 1984), the periods with enhanced OT actions (Hatton and Wang, 2008). The present study unambiguously verified that OT-reduced GFAP is directly mediated by astrocytic OTRs. The supportive evidences include that: (1) astrocytes in the SON express OTRs and OT but not VP elicited GFAP reduction under the same condition; (2) the effect of OT on GFAP did not require neuron-mediation since tetanus toxin could not block this OT action; and (3) OT-evoked depolarization of astrocytic membrane potential was blocked by the OTRA, even in the present of tetanus toxin; and most importantly, there was molecular association between GFAP and OTRs. One argument could be that tetanus toxin does not block the release of lipophilic compounds and gases, e.g., endocannabinoids (Hirasawa et al., 2004) and nitric oxide (Luckman et al., 1997), which may mediate OT actions. However, endocannabinoids (Aguado et al., 2006) and nitric oxide (Guo et al., 2007) increase but does not decrease GFAP expression. If their release is not blocked by tetanus toxin, OT-reduced GFAP expression (Wang and Hatton, 2009) should be weakened, even blocked but not increased. Thus, we conclude that OT can directly act on astrocytes and reduce GFAP expression via OTRs.

### OT Modulation of GFAP Metabolism and its Concentration- and Time-Dependence

Similar to those found in lactating rats (Wang and Hatton, 2009), OT concentration dependently reduced GFAP filaments in male rats. Interestingly, different concentrations of OT reduced GFAP likely via different metabolic processes. At lower levels, OT reduced both GFAP monomers at 50 kDa and their fragments, illustrating an increase in decomposition of GFAP molecules (Wang and Hatton, 2009). At higher levels, OT increased GFAP fragments while maintaining lower levels of GFAP monomers. The increased GFAP fragments did not contribute to the polymerization of GFAP filaments since GFAP polymerization is based on monomers but not their fragments. Moreover, effects of activating OTRs on GFAP levels are related to the time course of OT actions. In previous study, we showed that OT reduced GFAP protein and filaments in brain slices from lactating rats in a time-dependent manner (Wang and Hatton, 2009). This finding is confirmed in the present study that mSIRK-reduced GFAP expression (Figure 3) or OT-reduced astrocyte absorption of Bauer peptide (Figure 6) were also time-dependent in the SON of male rats. This is in agreement with the feature of activation of G Protein-coupled receptors and the relatively slow metabolic processes of GFAP.

### Intracellular Processes Following OTR Activation

In this study, we confirmed not only the expression of OTRs at astrocytes in the SON reported previously (Wang and Hatton, 2006), but also identified the mediation of Gβγ signaling in OT effects on SON functions and the mutually interactive processes between pERK 1/2 and PKA in OT-evoked GFAP plasticity.

In previous study on lactating rats, we found that OT-evoked activation of OT neurons was mainly mediated by Gβγ signaling but not Gα signaling following OTR activation of G_q/11_ type G protein (Wang and Hatton, 2007a). The present study extends this finding by showing that mSIRK evoked GFAP reduction (Figure 3) as OT did, which was in synergy with the expression of pERK 1/2 (Figure 4). Moreover, along with the activation of ERK 1/2 in 5 min and its reversal at 30 min (Wang and Hatton, 2007b), OT- and/or mSIRK-evoked reduction of GFAP as well as OT-modulated astrocytic absorption of Bauer-peptide became significant after 15–30 min. These processes were modulated not only by pERK 1/2 blockers but also by PKA-modulating agents. Lastly, at different stages of suckling stimulation that are associated with different levels of GFAP and pERK 1/2 expressions (Wang and Hatton, 2007b), cPKA (Figure 7) also had different expressions. Lastly, activating PKA with IPT or blocking ERK 1/2 phosphorylation decreased GFAP polymerization; blocking PKA blocked OT-evoked GFAP reduction while blocking ERK 1/2 activation simulated effects of OT on GFAP. Thus, both pERK1/2 and PKA are involved in OT-modulation of GFAP plasticity downstream to OTR-Gβγ signaling pathway.

In addition to these classical signaling processes, our finding also fills in a gap between OT-evoked GFAP plasticity and astrocytic functions by showing the depolarizing effects of OT on astrocyte membrane potentials and the OT-reduced absorption of Bauer Peptide. As shown in Figure 2, OT could depolarize astrocyte membrane potentials in 5–10 min of action. This could be an effect of OTR activation on the mobilization of IP3-sensitive intracellular Ca^2+^ stores (Di Scala-Guenot et al., 1994), one of the major downstream events of OTR activation. The increased intracellular Ca^2+^ levels could account for this initial depolarizing effect of OT because of its neutralization of intracellular negative charges. Moreover, this depolarization could account for the reduced absorption of Bauer Peptide at 30 min of OT treatment (Figure 6). Increased intracellular Ca^2+^ levels in astrocytes could cause hyper-phosphorylation of GFAP and its depolymerization (Heimfarth et al., 2016); the disruption of GFAP filaments could influence the expression of peptide transporter PepT2 (Dieck et al., 1999) due to losses of the scaffolding and guiding roles of GFAP (Hou et al., 2016; Wang and Parpura, 2016). As a result, astrocyte absorption of Bauer Peptide and the fluorescence intensity were also reduced. This proposal is supported by previous findings that reduction of GFAP also reduced its molecular association with glutamine synthetase (Wang et al., 2013b) and vesicular GABA transporters (Wang et al., 2013a) in astrocytes although directly assaying intracellular Ca^2+^ levels remains to be performed.

### Antagonistic but Coordinated Interaction between pERK 1/2 and PKA in OT-Modulation of GFAP Plasticity

Both pERK 1/2 and PKA are involved in OT-elicited GFAP reduction but play different roles. Activation of PKA can mediate OT-reduced GFAP filaments while certain level of basal PKA activity is necessary to maintain basal GFAP expression. In the presence of OT, KT5720 did not only increase GFAP filaments and 50 kDa GFAP, but also increased GFAP fragments. However, KT5720 itself reduced basal GFAP filaments. Thus, combining with Western blotting results, we propose that at resting conditions, PKA promotes GFAP polymerization by reducing dissembling or decomposition of GFAP monomers in the presence of pERK 1/2; when strongly activated, PKA causes GFAP depolymerization. The increased GFAP filaments by KT5720 to OT stimulation can be attributable to a simultaneously increased dissembling and polymerization of GFAP monomers by OT-increased pERK 1/2 (Wang and Hatton, 2007b).

It is clear that pERK 1/2 can promote dissembling of GFAP monomers since U0126 reduced GFAP fragments with or without OT. Thus, reduction of GFAP monomers by OT-activated pERK 1/2 may counterbalance, even overrule, PKA-increased GFAP monomers, accounting for the increases in GFAP fragments in response to KT5720 plus OT. Moreover, pERK 1/2 can promote GFAP polymerization despite its increasing the dissembling of GFAP monomers. This proposal is supported by the fact that inhibition of ERK 1/2 phosphorylation significantly reduced basal GFAP filaments and increased 50 kDa GFAP proteins. Alternatively, OT-elicited activation of PKA in the presence of U0126 can explain the decrease in GFAP fragments while increasing GFAP monomers. However, without pERK 1/2, even OT could still activate PKA, GFAP monomers failed to be polymerized into GFAP filaments. As a whole, the actions of these two protein kinases are antagonistic but coordinated in OT-modulated GFAP plasticity. This conclusion is in agreement with that PKA and pERK 1/2 play antagonistic roles in astrocytic proliferation (Bayatti and Engele, 2001) and in the OTR signaling (Zhong et al., 2003).

Further analysis highlights the possibility that there are spatiotemporally coordinative actions between the two kinases in OT-elicited GFAP reduction. It is clear, simply increasing cAMP or reducing pERK 1/2 levels did not elicit the spatial feature of GFAP plasticity observed in lactating rats. By contrast, using confocal microscopy, we found critical clues for a microdomain-specific regulation of the two kinases. During suckling, pERK 1/2 is translocated into somata (Wang and Hatton, 2007b), which indicates a separation of pERK 1/2 from the majority of GFAP in astrocytic processes. Following a reversal increase in GFAP upon occurrence of milk-ejection, pERK 1/2 was also expressed in astrocytic proximal processes (Wang and Hatton, 2007b).

**Figure 8:**
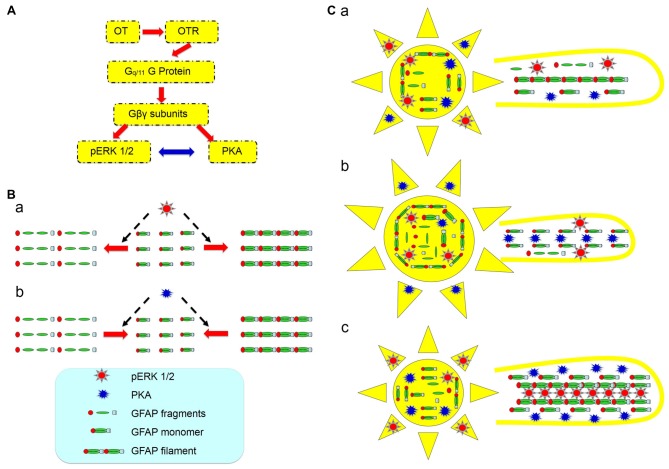
Diagram of hypothetic cellular and molecular mechanisms underlying OT modulation of GFAP plasticity in astrocytes. **(A)** OTR signaling in association with OT-evoked GFAP reduction. The red arrows show the flow of signaling transduction and the bidirectional blue arrow indicates a relationship of mutual inhibition. **(B)** Effects of activation of pERK 1/2 **(a)** and PKA **(b)** on GFAP metabolism/plasticity, respectively. **(C)** GFAP plasticity of astrocytic somata (left panels) and processes (right panels) in the SON at different stages of suckling at its differential associations with pERK 1/2 and PKA. **(Ca)** Before suckling; **(Cb)** during the first 5–10 min of suckling with the occurrence of milk ejection; and **(Cc)** 1 min within the occurrence of the third or fourth milk ejections.

A possible role for the increased PKA levels at the milk-ejection reflex is to provide GFAP monomers for pERK 1/2 to build new GFAP filaments at astrocytic processes. The expression of pERK 1/2 at somata (Wang and Hatton, 2007b) and activation of PKA at the processes could account for the GFAP increases in astrocytic somata and decreases in the processes at the initial stages of suckling (Figure 7), explaining the inhibitory effect of OT on GFAP expression. Noteworthy is that following the occurrence of the milk-ejection reflex, cPKA expression in the processes further increased from the elevated levels during suckling, which was accompanied with increased molecular association between GFAP and pERK 1/2 but decreased molecular association between GFAP and cPKA. It is likely that the increased cPKA provided GFAP monomers that are essential for pERK 1/2-evoked GFAP polymerization and filament formation; however, due to the reduced direct interaction of cPKA with GFAP, its depolymerization role gave the way to the polymerization role of pERK 1/2, and resulted in the increase in GFAP filaments and its ensuing expansion of astrocytic processes. Since GFAP filament is positively correlated to the extension of astrocyte processes (Pekny et al., 2007) and OT neuronal activity (Wang and Hamilton, 2009), pERK 1/2 and PKA modulated GFAP plasticity thus could determine OT neuronal activity under a variety of physiological and pathological conditions. Figure 8 is a schematic drawing of OTR signaling in OT-elicited GFAP plasticity.

## Conclusion

The present study revealed that OT-elicited GFAP plasticity is associated with sequential activation of ERK 1/2 and PKA via OTR signaling pathway in an antagonistic but coordinated manner with microdomain-specific features. This finding not only confirms that OT can efficiently facilitate neuronal activity by eliciting retraction of astrocyte processes (Wang and Hatton, 2009), but also provide a novel approach to alter astrocyte functions by directly changing GFAP plasticity through modulating the activity of pERK 1/2 and PKA. Since GFAP-associated astrocytic plasticity is extensively involved in physiological processes, such as OT secretion in lactation (Liu X. et al., 2016), reproduction (Liu X. Y. et al., 2016), and immunological activity (Wang et al., 2015; Li T. et al., 2017), as well as VP secretion in hyponatremia (Wang et al., 2011; Jiao et al., 2017) and ischemic stroke (Jia et al., 2016; Wang and Parpura, 2016) in addition to psychiatric disorders (Verkhratsky et al., 2016), neurodegenerative diseases (Vardjan et al., 2017) and many others (Yang et al., 2013; Pekny et al., 2016), further exploration of signaling process regulating GFAP plasticity is warranted.

## Author Contributions

PW and Y-FW performed the experiment and data analysis; PW wrote the first draft; DQ participated in discussion and revision; Y-FW designed the study and made the final revision.

## Conflict of Interest Statement

The authors declare that the research was conducted in the absence of any commercial or financial relationships that could be construed as a potential conflict of interest.
